# Cdc25A phosphatase: a key cell cycle protein that regulates neuron death in disease and development

**DOI:** 10.1038/cddis.2017.115

**Published:** 2017-03-23

**Authors:** Subhas Chandra Biswas, Priyankar Sanphui, Nandini Chatterjee, Stav Kemeny, Lloyd A Greene

**Affiliations:** 1 Cell biology and Physiology Division, CSIR-Indian Institute of Chemical Biology, Kolkata 700 032, India; 2 Department of Pathology and Cell Biology, Columbia University Medical Center, New York, NY 10032, USA

Cell cycle molecules are mostly dormant in differentiated neurons that are post-mitotic and in the G_0_ state of the cell cycle. However, a wealth of evidence strongly suggests that in response to a wide variety of apoptotic stimuli, including trophic factor deprivation, exposure to *β*-amyloid (A*β*) and DNA damage, neurons emerge from the G_0_ state with aberrant expression/activation of cell cycle proteins.^1^ This emergence is characterized by a consistent set of events related to the cell cycle that culminate in neuron death. Initial responses include activation of G1/S cyclin-dependent kinases (Cdks), such as Cdk4 that in turn phosphorylate retinoblastoma (pRb) family proteins and lead to dissociation of repressor complexes comprising E2F and pRb proteins, so that E2F-binding genes are de-repressed. Among genes that are de-repressed by loss of E2F-Rb family complexes are the B- and C-myb transcription factors that in turn transactivate Bim, a pro-apoptotic protein that promotes caspase activation and subsequent neuron death.^1, 2, 3, 4^ This set of events has been termed the ‘apoptotic cell cycle pathway'.

Cell division cycle 25A (Cdc25A), a member of a family comprising Cdc25A, B and C, is a dual specificity phosphatase that dephosphorylates inhibitory phosphates on adjacent threonine and tyrosine residues of Cdks such as Cdk4.^5^ This step is essential for initiation of cell cycle in proliferating cells. However, it was not known whether in the non-dividing neurons, the same events would activate the apoptotic cell cycle pathway. In our recent paper published in *Cell Death Discovery*,^6^ we report several novel findings regarding the potential role of Cdc25A in neuron death. First, Cdc25A is required for activation of the apoptotic cell cycle pathway and neuron death in response to nerve growth factor (NGF) deprivation and A*β* treatment. Second, Cdc25A acts upstream of Cdk-mediated Rb phosphorylation and caspase-3 cleavage. Third, NGF deprivation and A*β* lead to rapid increases in Cdc25A mRNA and protein levels. NGF withdrawal causes an increase in Cdc25A activity as well. These events occur at about the same time that apoptotic insults lead to Cdk4 activation and Rb phosphorylation in our experimental systems and well precede evident signs of neuron death.

To follow-up on our findings that NGF deprivation and A*β* induce Cdc25A expression in neurons, we studied the mechanism by which this occurs. This revealed a pathway in which NGF deprivation or A*β* treatment leads successively to Akt inactivation, FoxO activation, and suppression of miR-21 levels with consequent elevation of Cdc25A. Normally, Akt phosphorylates FoxO transcription factors, which limits them to the cytosol (Figure 1). A*β* treatment inhibits Akt signalling in neurons and Akt activity is diminished in brains of AD patients and of APP transgenic mice.^7, 8^ NGF deprivation also rapidly decreases Akt phosphorylation/activity.^9^ When Akt signalling is suppressed,^10^ FoxO proteins are dephosphorylated and translocate to the nucleus where they are transcriptionally active.^3, 8, 9^ We show that FoxO3a downregulates miR-21 (a microRNA that usually suppresses Cdc25A expression) thereby upregulating Cdc25A in A*β*-treated neurons.^6^ Moreover, a miR-21 mimic was sufficient to block Cdc25A mRNA and protein induction by A*β*. Thus our study places Cdc25A upstream of Cdk4 activation and subsequent events in the apoptotic neuronal death pathway and identifies a set of signaling events by which NGF deprivation or A*β* exposure regulate cellular Cdc25A levels and activity, leading to neuron death (Figure 1).

A previous study reported that camptothecin-induced DNA damage in cultured neurons activates Cdc25A and that inhibition or knockdown of Cdc25A blocks Cdk4 activation and Rb phosphorylation as well as cell death, thus linking Cdc25A to the apoptotic cell cycle pathway.^11^ However, in contrast to A*β* or NGF deprivation, camptothecin did not change Cdc25A levels (and therefore not likely the FoxO-miR21 pathway), but rather was correlated with loss of activity of the checkpoint 1 kinase (Chk1).^11^ Therefore, although it appears that distal effectors of the neuronal apoptotic cell cycle pathway are similar for different apoptotic stimuli, multiple mechanisms may exist to initiate the pathway via Cdc25A.

Ours is the first report to identify Cdc25A as a required upstream activator of the apoptotic cell cycle pathway in trophic factor-deprived neurons and that its levels after A*β* treatment are elevated by a pathway involving FoxOs and miR-21.^6^ In the case of A*β*, our findings are consistent with the report that neurons in post-mortem brains from AD patients have elevated Cdc25A levels and that brain tissue from AD patients has higher Cdc25A phosphatase activity compared to non-AD brains.^12^ Also, Kruman *et al.*^13^ described a 3–4-fold increase in Cdc25A protein in A*β*-treated cortical neuron cultures. Thus, our study favours a mechanism in which A*β* elevates Cdc25A expression via FoxO-miR21 signalling and our data clearly identify Cdc25A as a required player in A*β*-induced neuron death.

In summary, our study reveals that Cdc25A is elevated, activated and has an essential role in neuronal cell death evoked by apoptotic stimuli relevant to normal development and to AD. Because Cdc25A is an inhibitable enzyme, our study identifies Cdc25A as a potential target to block pathologic neuron degeneration and death in AD and other pathologies in which the neuronal apoptotic cell cycle pathway is activated. In support of this idea, a selective Cdc25A inhibitor has been shown to be effective in several non-neuronal experimental disease models and without reported toxicity.^14^

## Figures and Tables

**Figure 1:**
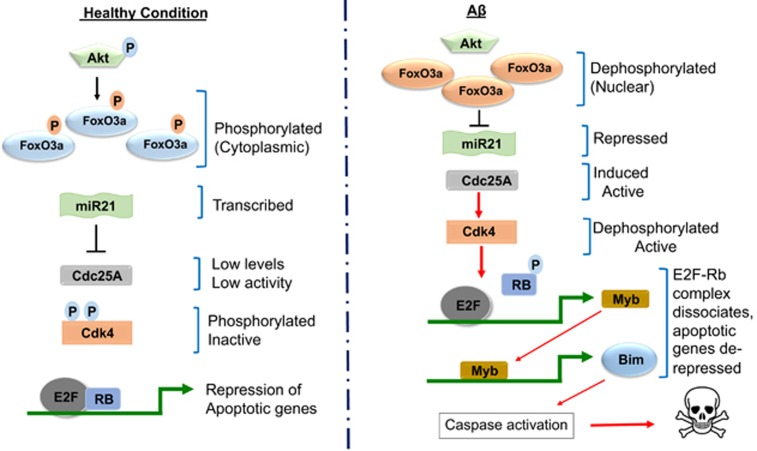
Scheme depicting a molecular pathway by which Cdc25A is induced/activated and promotes neuron death in disease and development. In healthy cells, Akt phosphorylates FoxO transcription factors and retains them in the cytosol. miR-21, a microRNA that suppresses Cdc25A expression and that is negatively regulated by FoxO3a, remains elevated in the nucleus to block the apoptotic cell cycle pathway. A*β* treatment and NGF deprivation inhibit neuronal Akt signalling. When Akt signalling is suppressed, FoxO proteins are activated and translocate to the nucleus. FoxO3a downregulates miR-21 and thereby upregulates Cdc25A. Elevated and activated Cdc25A leads to Cdk4 activation and subsequent Rb phosphorylation, expression of E2F-responsive genes such as B- and C-myb, induction of Bim, caspase activation and neuron death
